# Impact of frailty on outcomes of pancreatic surgery: a systematic review and meta-analysis

**DOI:** 10.3389/fnut.2026.1706900

**Published:** 2026-03-02

**Authors:** Hefang Li, Mouchun Gong, Kehui Chen, Yaru Huang

**Affiliations:** Department of General Surgery, The First People’s Hospital of Lin’an District, Hangzhou (Lin’an People’s Hospital Affiliated to Hangzhou Medical College), Hangzhou, Zhejiang, China

**Keywords:** complications, frail, geriatric, mortality, pancreatectomy

## Abstract

**Objective:**

Frailty is being recognized as a risk factor for adverse outcomes after various surgical procedures. However, its impact on pancreatic surgery outcomes remains uncertain. We hereby reviewed evidence on the difference in the risk of complications and mortality between frail and non-frail patients undergoing pancreatic surgery.

**Methods:**

PubMed, Embase, Scopus, and Web of Science were searched from 1^st^ January 1980 to 17^th^ June 2025, for relevant studies. The endpoints of the study were all complications, Clavien-Dindo grade ≥3 (CD ≥ 3) complications, reoperation, and readmissions. Random-effect meta-analysis was conducted.

**Results:**

A total of 11 studies were included. A pooled analysis revealed that frailty was associated with an increased risk of all complications in patients undergoing pancreatic surgery (OR: 1.51, 95% CI: 1.01–2.24, I^2^ = 61%). Meta-analysis showed no significant difference between frail and non-frail patients for CD ≥ 3 complications (OR: 1.34, 95% CI: 0.86, 2.09 I^2^ = 31%), readmission (OR: 1.41, 95% CI: 0.75, 2.62 I^2^ = 0%) and reoperation (OR: 2.32, 95% CI: 0.63, 8.50 I^2^ = 0%). However, frail patients had a significantly higher risk of short-term (OR: 2.54, 95% CI: 1.39, 4.61, I^2^ = 50%) mortality as compared to non-frail patients after pancreatic surgery. Sensitivity and subgroup analysis generated mixed results.

**Conclusion:**

The presence of preoperative frailty may lead to increased risk of short-term mortality and morbidity in patients undergoing pancreatic surgery. Frailty was not associated with major complications, readmission, or reoperations. However, results must be interpreted with caution owing to limited data and high inter-study heterogeneity.

**Systematic review registration:**

https://www.crd.york.ac.uk/prospero/, CRD420251049842.

## Introduction

With an estimated 495,773 new cases in 2020, pancreatic cancer is among the most prevalent malignancies worldwide. It is also the sixth largest cause of cancer-related deaths globally, accounting for an estimated 466,003 fatalities (1). Despite improvements in cancer care, pancreatic cancer remains one of the solid cancers with the worst prognosis, with only about 9% of people surviving after 5 years (2). Its prevalence is rising in Western countries and is anticipated to become the second biggest cause of cancer deaths in a decade (3). Age is a major risk factor for pancreatic cancer, with 85% of patients aged over 60 and an average age of 71 at diagnosis. Age not only increases the incidence but is also associated with a worse prognosis (4, 5).

Surgical excision is the only possibly curative treatment for nonmetastatic malignancy. Due to the prevalence of locally advanced or metastatic disease, only 15–20% of patients are eligible for surgery at diagnosis (2). Recent advancements in surgery, including minimally invasive procedures, better perioperative care, and centralization in high-volume institutions, have resulted in improved postoperative results in recent years (6, 7). Nevertheless, significant morbidity and mortality are still associated with pancreatic surgery, making it a high-risk procedure (8). Although several studies suggest that even elderly patients can withstand major pancreatic surgery, selecting patients for treatment remains challenging (9, 10). Pancreatic cancer in older adults can cause nonspecific symptoms such as diminished mobility, cognitive decline, depressive disorders, malnutrition, and functional dependence. With advancing age, population variability becomes more apparent, and patients may develop comorbidities and frailty alongside their primary malignancy (11).

Frailty is characterized by multisystem decline in the physiological reserve capacity and susceptibility to stressors, which increases the risk of adverse health consequences, disability, and death. More common in the elderly, frailty results in low nutritional status, reduced mobility, decreased physical strength and muscle power, poor endurance, and impaired balance and cognitive function (12, 13). Frail patients have a shorter life expectancy and a higher risk of complications following surgery than those who are fit and of the same age (14). Despite being a disease of the elderly, there seems to be a scarcity of high-quality evidence on the impact of frailty in patients undergoing pancreatic surgery. Previously, Zhang et al. (15) in a meta-analysis have shown that about 45% of pancreatic cancer patients are frail and frailty is an independent risk factor for poor overall survival. However, their study included pancreatic cancer patients treated with multimodal therapies and did not focus on surgical complications. With no review examining the effect of frailty on surgical outcomes, we conducted the present study to compare the risk of complications and mortality between frail and non-frail patients undergoing pancreatic surgery.

## Materials and methods

We followed the Preferred Reporting Items for Systematic Reviews and Meta-Analysis (PRISMA) statement for the review (16). The meta-analysis protocol was assigned number CRD420251049842 on PROSPERO. The original protocol included only mortality and complications as the review outcomes. The only deviation from the protocol was the inclusion of reoperation and readmission as additional outcomes.

### Eligibility criteria

The inclusion criteria for the study were established using the PECO (Population, Exposure, Comparison, Outcome) framework. Studies that were conducted on a “Population” of patients undergoing pancreatic surgery were included. Studies were to compare frail patients with a cohort of non-frail individuals and use frailty as the “Exposure” variable, as measured on any standardized scale. Frailty was defined as a clinically recognizable state of increased vulnerability resulting from aging-associated decline in reserve and function across multiple physiologic systems such that the ability to cope with everyday or acute stressors is comprised (12).

The ‘Outcomes’ of the studies were to include mortality, complications, readmission, or reoperation. The language of publication and the design of the study were not restricted.

We excluded studies that focused on frail patients without a control group, did not report distinct data for pancreatic surgery, used surrogate markers of frailty (such as sarcopenia), and were conference abstracts only.

### Search and selection of studies

Two independent reviewers (MG & HL) conducted a thorough search of the PubMed, Embase, Scopus, and Web of Science literature databases. Eligible studies were those published between 1st January 1980 and 17th June 2025. The search queries that were created for the databases are presented in Supplementary Table 1. The references of all included articles were screened to identify additional relevant investigations. Additionally, a Google Scholar search was implemented to identify publications in the gray literature.

Duplicate studies were eliminated after the database-searched articles were imported into EndNote version X9 (Thomson Reuters, New York, NY, USA). The same two evaluators subsequently conducted a separate examination of the studies to ascertain whether they could be included in the review. This was accomplished by conducting a thorough analysis of the abstracts and titles of the articles. Subsequently, a comprehensive text analysis was performed on pertinent studies that were identified by either reviewer before they were included. Ultimately, the two reviewers’ disagreements were resolved through a discussion with a third reviewer (KC).

### Data extraction

Data extraction was performed by two reviewers (KC & YH) and cross-checked by a third reviewer (MG). Information obtained from the studies included details like author, location and study design, inclusion population, sample size, age, gender, body mass index (BMI), comorbidities, primary pathology, frailty index, cut-off used, prevalence of frailty, follow-up and outcomes assessed.

The endpoints of the study were all complications, Clavien-Dindo grade ≥3 (CD ≥ 3) complications, reoperation, and readmissions. Mortality data were further categorized into short-term mortality (up to 3 months) and long-term mortality (more than 1 year).

### Risk of bias analysis

Applying the Newcastle Ottawa Scale (NOS) (17), the two reviewers (HL & KC) gave a quality score to each article (varying from zero to nine). The evaluation was conducted in three distinct areas: exposure or outcome identification, comparability of groups, and participant selection. The maximum possible points for each of the three categories are four, two, and three, respectively. To resolve any discrepancies, the third author (YH) was consulted. Studies were graded as high (score 8–9), medium and (score 6–7) low quality (<6). Points for comparability of groups were given based on the adjustment of confounders. In studies that did not report adjusted data, zero points were awarded. Points for follow-up (Outcome assessment) were awarded only when studies reported at least 6 months of follow-up data.

### Data analysis

Data were pooled using the DerSimonian and Laird random-effects meta-analysis model, which accounts for both within-study and between-study variations. This model was chosen as it provides a conventional estimate of between-study variance. Given the heterogeneity across included studies, this approach was considered appropriate and has been widely applied in surgical outcome research. Odds ratios (OR) with 95% confidence intervals (CI) were either extracted from the studies or calculated from raw data. If adjusted data were available, it was preferred for extraction. We combined both adjusted and unadjusted data, and the impact of unadjusted data was examined through subgroup analysis. Meta-analysis was performed if at least two studies reported the outcome. Pooled results were presented as ORs. Summary measures were displayed in forest plots, where the size of each square data marker indicates the inverse variance of the natural logarithm of the OR from each study. The diamond indicates the pooled OR.

Statistical significance was considered for *p*-values less than 0.05. Data analysis was conducted in “Comprehensive Meta-analysis” software (Version 3, Biostat, Englewood, NJ, USA). Heterogeneity among studies was assessed through Cochran’s Q statistic and the I^2^ index. I^2^ of over 50% and/or *p* < 0.05 indicated significant heterogeneity. Egger’s test and funnel plots were used to assess publication bias. Sensitivity analysis was conducted by removing one study at a time from the meta-analysis to evaluate the influence of various exclusions on the combined OR (only for complications and short-term mortality, as limited studies were available for other outcomes). Subgroup analysis was conducted based on study design, location, frailty assessment tool, and type of data (univariate or adjusted). Raw data of the study is available from the corresponding authors on reasonable request.

## Results

### Search results

**Figure 1:**
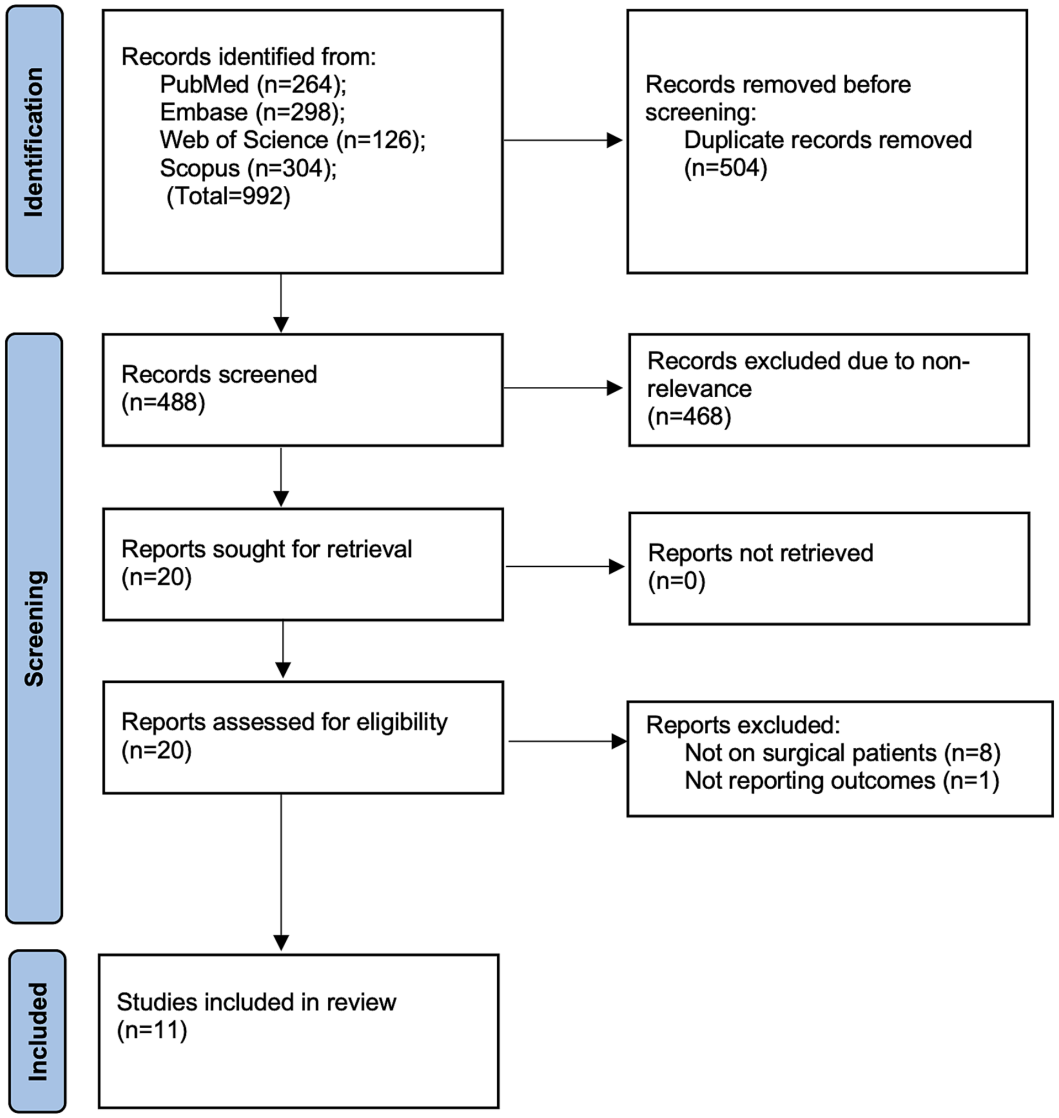
Study flowchart.

The number of studies retrieved from the databases and screened at each stage is depicted in the PRISMA flowchart of the study (Figure 1). Overall, 992 studies were available based on the keywords. After deduplication, 488 records were available, of which 20 were deemed related to the review question by the two reviewers. After a comprehensive full-text assessment, 11 studies (18–29) were included in the review. The reviewers did not identify any points of disagreement regarding the inclusion or exclusion of any study. Moreover, no additional studies were found in the reference lists of the included articles or in Google Scholar.

### Baseline details

The data retrieved from the articles are shown in Table 1. The studies were published between 2018 and 2025. Regarding study design, two were prospective, while the rest were retrospective. All studies except three originated from the USA or Japan; the remaining three were from the Netherlands, Italy, and Canada. The sample sizes varied widely, from 52 to 16,028 patients. The combined sample size of the studies was 18,013, with the majority of patients in the elderly age group. The included population was predominantly elderly, with mean or median ages ranging from 63 to 75 years, and the proportion of males varied from 40.8 to 68.2%. When reported, the mean BMI ranged between 21.7 kg/m^2^ and 27.1 kg/m^2^. Reporting of comorbidities such as diabetes mellitus, hypertension, and cardiovascular disease was inconsistent across studies, although several documented a high prevalence of diabetes and hypertension (up to 69.8%). Most patients underwent pancreaticoduodenectomy or other forms of pancreatic resection for pancreatic ductal adenocarcinoma, which was the primary underlying pathology in nearly all studies. Only a few included patients with benign pancreatic lesions or non-pancreatic ductal adenocarcinoma malignancies. In the frailty assessment tools used by the studies included the 5-point modified frailty index (mFI-5), 11-item modified frailty index (mFI-11), Comprehensive Geriatric Assessment (CGA), Clinical Frailty Scale (CFS), and the Japanese version of the cardiovascular health study criteria. Prevalence of frailty varied from 10 to 74%. Seven studies reported only in-hospital outcomes. Follow-up of the remaining studies ranged from 3 months to 2 years.

**Table 1:** Details of included studies.

Study	Location	Design	Included population	Sample size (n)	Males (%)	Age (years)	BMI (kg/m^2^)	DM (%)	HT (%)	CVD (%)	Primary pathology- %	Frailty index and cut-off	Frail patients (%)	Outcomes assessed	Follow-up
Kwon 2025	USA	P	Robotic PD surgery for benign and malignant tumors	116	44	70.7	26.8 ± 4.8	45.7	69.8	15.5	PDAC- 71.6	mFI-5 ≥ 2	45.7	All complications	3 months
Khalid 2024	USA	R	PDAC patients undergoing PD	250	49.2	74.6	25.7 ± 5	NR	NR	NR	PDAC- 100	mFI-5 > 2	10	All complications, CD > 3 complication, mortality, readmission	3 months
Hartog 2024	Nether lands	P	≥70 years with pancreatic malignancy undergoing surgery	88	68.2	75	25.1 [22.1–27.1]	33	44.3	21.6	PDAC-76.1	CGA	74	All complications, CD > 3 complication, mortality, readmission	25 months
Onji 2023	Japan	R	Pancreatic malignancy undergoing surgery	52	50	72	22.2 [21.3–24.2]	57.7	NR	17.3	NR	CFS ≥ 4	28.8	All complications	In-hospital
Paiella 2022	Italy	R	≥70 years with pancreatic malignancy undergoing distal pancreatectomy	204	47	74	25 ± 4	NR	NR	NR	PDAC-58	mFI-5 ≥ 1	74	All complications, CD > 3 complication, mortality, readmission, reoperation	In-hospital
Li 2022	Canada	R	Patients undergoing PD	554	59	67	26 [17–54]	NR	NR	NR	PDAC-50	11-mFI ≥ 0.27	12	All complications, CD > 3 complication, mortality	3 months
Cramer 2022	USA	R	Patients undergoing pancreatic surgery	1,266	50	NR	27.1 ± 5.6	NR	NR	NR	PDAC-56	mFI-5 ≥ 2	NR	All complications, mortality, readmission, reoperation	In-hospital
Yamada 2021	Japan	R	PDAC patients undergoing PD	120	40.8	71	NR	48.3	NR	NR	PDAC-100	CFS ≥ 4	24.2	All complications	In-hospital
Mima 2021	Japan	R	Patients undergoing pancreatic resection	142	56	NR	NR	42	NR	NR	PDAC-94	CFS ≥ 4	20.4	CD > 3 complication	In-hospital
Nakano 2020	Japan	R	Patients undergoing curative pancreatectomy	93	61.3	72	21.7 [14.2–33]	25.9	NR	NR	Pancreatic cancer-49.5	J-CHS criteria	11.8	CD > 3 complication	In-hospital
Guyton 2018	USA	R	Patients undergoing pancreatic resection	16,028	48.2	63.4	NR	NR	NR	NR	NR	mFI ≥ 2	18.7	Mortality	In-hospital

PD, pancreaticoduodenectomy; PDAC, pancreatic ductal adenocarcinoma; DM, Diabetes mellitus; HT, hypertension; CVD, cardiovascular disease; n, number; mFI-5, 5-factor modified frailty index; NR, not reported; P, prospective; R, retrospective; CGA, comprehensive geriatric assessment; CFS, clinical frailty scale; J-CHS, Japanese version of the cardiovascular health study; CD, Calvein Dindo.

### Risk of bias

Based on the author’s judgment, all studies were awarded NOS points as depicted in Table 2. All studies received four points for cohort selection. However, three studies received no points for comparability, as they reported only unadjusted data. For outcome assessment, one study received three points, which reported long-term follow-up, while all others received two points since they reported only in-hospital outcomes. Overall, three studies got six points (medium quality), while all others got eight or nine points (high quality).

**Table 2:** Risk of bias analysis.

Study	Selection of cohort	Comparability	Outcome assessment	Total NOS score
Kwon 2025	4	2	2	8
Khalid 2024	4	2	2	8
Hartog 2024	4	2	3	9
Onji 2023	4	2	2	8
Paiella 2022	4	0	2	6
Li 2022	4	2	2	8
Cramer 2022	4	2	2	8
Yamada 2021	4	0	2	6
Mima 2021	4	0	2	6
Nakano 2020	4	2	2	8
Guyton 2018	4	2	2	8

NOS, Newcastle Ottawa scale.

### Meta-analysis

**Figure 2:**
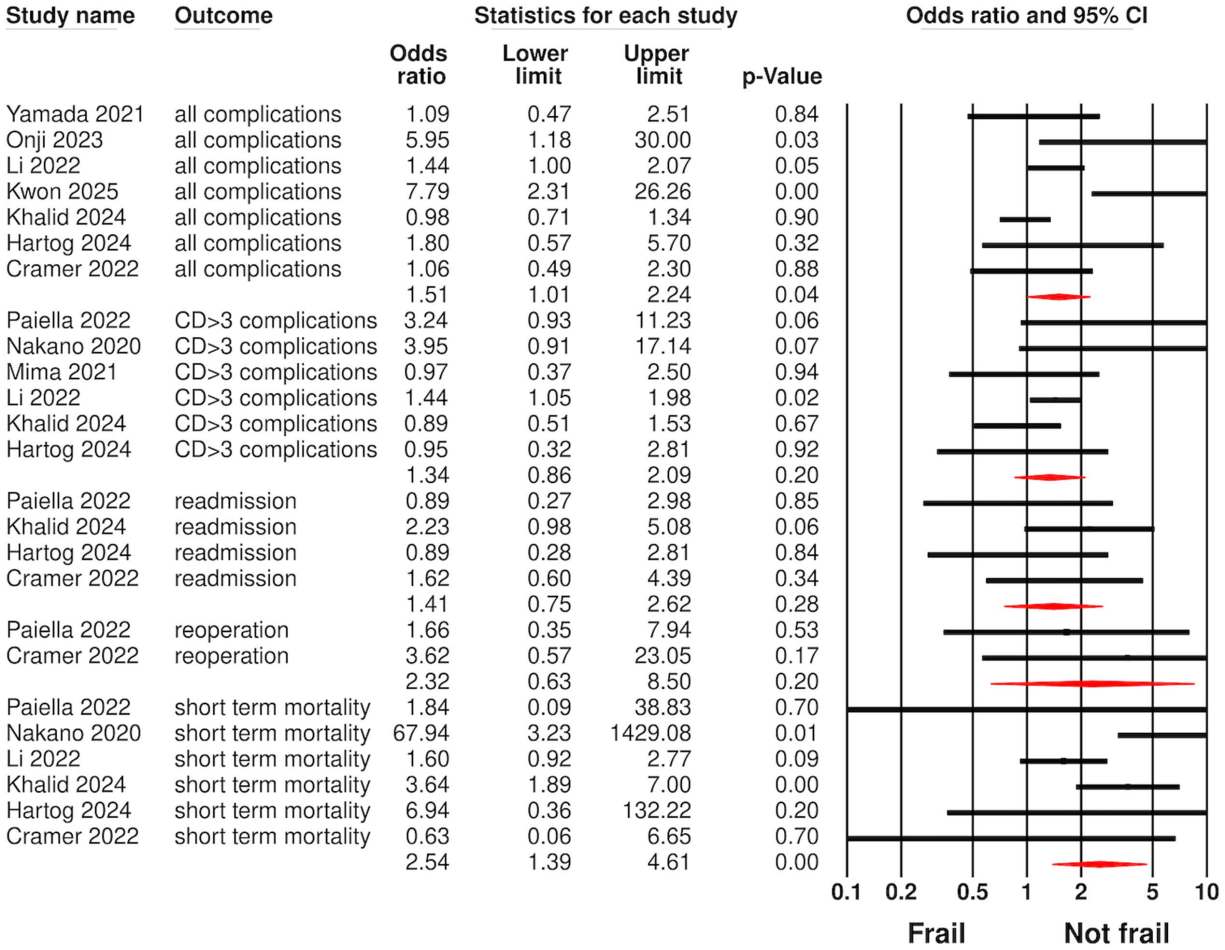
Meta-analysis of outcomes between frail and non-frail patients undergoing pancreatic surgery.

Figure 2 displays the meta-analysis of all outcomes examined in the review. Seven studies provided data on all complications, and a pooled analysis indicated that frailty was associated with a modest increase in the risk of all complications in patients undergoing pancreatic surgery (OR: 1.51, 95% CI: 1.01, 2.24, I^2^ = 61%). For CD ≥ 3 complications, data were reported by six studies, and meta-analysis showed no significant difference between frail and non-frail patients (OR: 1.34, 95% CI: 0.86, 2.09, I^2^ = 31%). Six studies supplied data on short-term mortality. Pooled analysis revealed that frail patients had a significantly greater risk of short-term (OR: 2.54, 95% CI: 1.39, 4.61, I^2^ = 50%) mortality compared to non-frail patients after pancreatic surgery. However, the risk of readmission (OR: 1.41, 95% CI: 0.75, 2.62, I^2^ = 0%) and reoperation (OR: 2.32, 95% CI: 0.63, 8.50, I^2^ = 0%) did not differ between the two groups. Only one study provided data for long-term mortality. Hartog et al. (27), with a median follow-up of 25 months, reported that frail patients had a significantly higher risk of mortality than non-frail patients [hazard ratio (HR): 3.36, 95% CI: 1.43, 7.89, *p* = 0.006] after adjustment for age, sex, and tumor location.

### Publication bias and sensitivity analysis

Egger’s test for all outcomes was non-significant (*p* > 0.05) and funnel plots (Supplementary Figures 1–4) showed no gross asymmetry, indicating no publication bias. Sensitivity analysis was conducted for complications and short-term mortality. Results are shown in Supplementary Figures 5–7. The results of all complications became non-significant after following the exclusion of several studies in the sensitivity analysis. For short-term mortality and CD > 3 complications, the results were non-significant after excluding Khalid et al. (26).

### Subgroup analysis

The results of the subgroup analyses are presented in Table 3. For all complications, the results remained non-significant in all subgroup analyses based on study location, frailty tool, and type of data. However, results were significant for prospective studies (OR: 3.63, 95% CI: 1.44, 9.14, I^2^ = 66%). On the other hand, the results of CD ≥ 3 complications remained non-significant for all subgroup analyses based on study location, design, frailty tool, and type of data. Subgroup analyses for short-term mortality revealed significant results for Japanese (one study) and North American studies, but not for European studies. The results were also found to be significant for retrospective studies (OR: 2.57, 95% CI: 1.07, 6.15, I^2^ = 58%) but not for the lone prospective study. Based on frailty assessment tool, results were significant for studies using J-CHS and mFI-5 criteria, but not for those using 11-mFI and CGA. We also noted significant results for studies reporting unadjusted data (OR: 9.49, 95% CI: 1.51, 59.65, I^2^ = 57%) but not for those reporting adjusted data.

**Table 3:** Subgroup analysis results.

Outcome	Variable	Groups	Studies	Odds ratio	I^2^
All complications	Location	Europe Japan North America	1 2 4	1.80 95% CI: 0.41, 7.96 1.85 95% CI: 0.65, 5.25 1.50 95% CI: 0.85, 2.64	0 70 74
	Study design	Prospective Retrospective	2 5	3.63 95% CI: 1.44, 9.14 1.24 95% CI: 0.85, 1.80	66 38
	Tool	11-mFI CFS CGA mFI-5	1 2 1 3	1.44 95% CI: 0.30, 6.99 2.07 95% CI: 0.52, 8.25 1.80 95% CI: 0.26, 12.30 1.72 95% CI: 0.64, 4.68	0 70 0 80
	Data	Univariate Adjusted	2 5	1.34 95% CI: 0.52, 3.43 1.66 95% CI: 0.98, 2.81	0 73
CD > 3 complications	Location	Europe Japan North America	2 2 2	1.67 95% CI: 0.54, 5.26 1.67 95% CI: 0.53, 5.27 1.16 95% CI: 0.62, 2.19	53 60 56
	Study design	Prospective Retrospective	1 5	0.95 95% CI: 0.21, 4.30 1.38 95% CI: 0.90, 2.11	0 42
	Tool	11-mFI CFS CGA J-CHS mFI-5	1 1 1 1 2	1.44 95% CI: 0.31, 6.78 0.97 95% CI: 0.16, 5.79 0.95 95% CI: 0.15, 6.12 3.95 95% CI: 0.48, 32.60 1.50 95% CI: 0.43, 5.19	0 0 0 0 71
	Data	Univariate Adjusted	3 3	1.34 95% CI: 0.79, 2.26 1.39 95% CI: 0.71, 2.73	55 28
Short term mortality	Location	Europe Japan North America	2 1 3	3.65 95% CI: 0.41, 32.76 67.94 95% CI: 2.91, 1586.67 2.13 95% CI: 1.08, 4.22	0 0 56
	Study design	Prospective Retrospective	1 5	6.94 95% CI: 0.28, 172.98 2.57 95% CI: 1.07, 6.15	0 58
	Tool	11-mFI CGA J-CHS mFI-5	1 1 1 3	1.6 95% CI: 0.79, 3.23 6.94 95% CI: 0.35, 136.53 67.94 95% CI: 3.13, 1474.20 2.98 95% CI: 1.44, 6.17	0 0 0 4
	Data	Univariate Adjusted	3 3	9.49 95% CI: 1.51, 59.65 2.08 95% CI: 0.94, 4.64	57 28

CGA, comprehensive geriatric assessment; CFS, clinical frailty scale; J-CHS, Japanese version of the cardiovascular health study; mFI-5, 5-factor modified frailty index; CD, Calvein Dindo; CI, confidence intervals.

## Discussion

The presence of preoperative frailty is a significant challenge during surgical intervention for different diseases. It is frequently linked to poorer postoperative outcomes, including increased overall complications, hospital readmissions, and markedly elevated mortality rates. The significance of frailty is underscored in contemporary times due to its rising prevalence. According to one long-term study of primary care data, the prevalence of frailty increased from 26.5 to 28.9% between 2006 and 2017 (30). Despite the increased prevalence, the impact of frailty on patients undergoing pancreatic surgery remains unclear. To the best of our knowledge, the present study is the first to pool evidence on the effect of frailty on complication, mortality, readmission, and reoperation rates after pancreatic surgery.

The meta-analysis showed that frailty was a predictor of both all complications and short-term mortality in patients undergoing pancreatic surgery, but did not predict CD ≥ 3 complications. The pooled estimates suggest that the presence of frailty was associated with a clinically significant 2.5 times increased risk of mortality. There was also a tendency for a modest increase in the risk of all complications (by 1.5 times) with the presence of frailty; however, the results were non-significant for major complications (CD ≥ 3), readmission, and reoperation. This variation might stem from different frailty assessment tools and their cutoff points, along with inconsistencies in how studies report and grade postoperative events. Frailty’s effect on overall complications could be partly confounded by pre-existing health conditions and nutritional issues, which increase susceptibility to less severe but frequent postoperative events without necessarily leading to severe complications. Additionally, the limited statistical power in some studies may have reduced the ability to detect true associations.

These results are generally similar to those of other meta-analysis studies reporting the effects of frailty on patients undergoing gastrointestinal surgery. Zhang et al. (31) in a recent meta-analysis of 18 studies noted that frailty was associated with an increased risk of major complications and 90-day mortality after hepatectomy, along with a tendency toward higher 30-day mortality. Niknami et al. (32) collated data from nine studies and found that frailty led to significantly higher short-term mortality and morbidity in patients undergoing cholecystectomy. Zhou et al. (33) combined data from 24 studies to show that frailty was a predictor of mortality, all complications, CD ≥ 3 complications, reoperation, readmission, blood transfusion, and prolonged hospitalization in patients undergoing colorectal cancer surgery. Another pooled analysis has demonstrated that higher mFI scores were linked with poor postoperative outcomes and worse overall survival in patients undergoing gastrectomy for gastric cancer (34). Indeed, the majority of prior meta-analysis studies have found an association between frailty and major complications. The non-significant results noted for CD ≥ 3 complications in our review were influenced by the study of Khalid et al. (26), the exclusion of which demonstrated significant results. The low prevalence of frailty in Khalid et al. (26) study could be one reason for the non-significant associations. Moreover, pancreatic surgery differs significantly in terms of physiological stress and postoperative recovery. Compared to hepatectomy or colorectal procedures, pancreatic resection typically involves longer surgical durations, increased intraoperative blood loss, and more complex anastomoses. Complications such as pancreatic fistula, delayed gastric emptying, and biliary leakage tend to be procedure-specific (7). They may be more affected by surgical technique and anatomy than by patient frailty. Moreover, patients undergoing pancreatic surgery often suffer from malnutrition related to malignancy, cholestasis, or systemic inflammation, which can mask the influence of frailty (9). These unique physiological and technical factors partly explain why, in our analysis, frailty was associated with overall morbidity and mortality, but not with major complications (CD ≥ 3).

High heterogeneity was noted in the meta-analyses of all complications and short-term mortality. This could be primarily attributed to variations in study designs, included populations, cancer stage, comorbidities, frailty assessment tools, frailty prevalence, surgical protocols, etc. Since detailed data on several such confounders were not available, we were unable to perform a comprehensive subgroup analysis. However, the subgroup analyses in the review produced mixed results, with some subgroups showing significant associations while others did not, suggesting cautious interpretation. These differences are most likely due to variations in study design, sample size, and statistical power rather than actual effect modification. Some subgroups, especially prospective studies and specific frailty assessment tools, were represented by only one or two small studies, leading to wide confidence intervals and limited accuracy. Although prospective studies indicated a stronger link between frailty and overall complications, this was based on few studies and might reflect the more systematic monitoring typical of prospective designs rather than a truly larger effect. Conversely, larger retrospective studies with greater statistical power may have underreported complications because they rely on administrative or registry data, which may weaken the observed associations. Importantly, although statistical significance varied across subgroups, the overall effect direction remained consistent, with frailty generally associated with poorer postoperative outcomes. Larger, well-powered prospective studies that use standardized definitions of frailty and outcome measures are necessary to determine if genuine subgroup-specific differences are present.

Variations in the tools used for frailty assessment are a significant limitation of the current review. The included studies used five different tools, and even with similar tools, minor variations in the cut-offs to define frailty were noted. Indeed, the statistical measurement of frailty remains a contentious issue across various specialties (35). As it offers a more dependable assessment of a patient’s biological age compared to chronological age alone, there is a need for a gold-standard criterion to more precisely categorize patients as frail, particularly in light of the increasing elderly population within surgical oncology practice (36, 37). Since the development of the “Fried Frailty Phenotype” scale in 2001, more than 70 tools have been created to assess frailty (37). The most commonly used tool in the included studies was the mFI, either in the form of mFI-5 or the more detailed version 11-mFI. The mFI-5, while convenient, may oversimplify frailty by focusing solely on five indicators. Simplifying a patient’s health status may lead to overlooking important features and compromising surgical outcomes. Notwithstanding this simplification, mFI-5 has demonstrated a strong association (Spearman coefficients > 0.9) with the 11-item index (38). However, there are differences between mFI and other scales, such as CFS and CGA. The CFS is a 9-point scale that offers a clinician-based evaluation of overall fitness or frailty, whereas the mFI diagnoses frailty based on the presence of certain health conditions. Evidence suggests that CFS may be a better predictor of frailty than mFI (39). CGA, on the other hand, is a detailed multidisciplinary diagnostic process that assesses the functional status, cognition, emotional status, nutritional status, comorbid medical conditions, drug history, and geriatric syndromes of the individual to eventually classify as frail. When it comes to risk stratification, CGA is difficult to interpret and resource-intensive, which limits its routine use (40). Given the scarcity of research comparing frailty assessment methods for pancreatic surgery patients, it is unclear at this time which instrument can better predict frailty state, and thus complications and mortality.

Various other limitations exist in the present review. The predominance of retrospective study designs inherently restricts our capacity to establish causality. The presence of selection bias cannot be negated, as retrospective data reflects biased clinical decisions. Another drawback is the generalizability of the findings. The majority of the studies came from two countries: Japan and the USA. Our findings may not apply to patients from diverse regions with varying demographics, healthcare access, and comorbidities. We could not assess the influence of essential confounders, such as the invasiveness of the surgery, surgeon experience, adjuvant therapies, and perioperative protocols, all of which could have contributed to variations in the risk of mortality and complications. Data on individual complication rates and long-term mortality were also scarcely reported by the studies, which limited a detailed analysis.

The reported findings also have clinical implications. Surgeons can identify high-risk candidates by using frailty indices and calculators to evaluate each patient’s health status before pancreatic surgery. It can also help them adjust and improve elements that contribute to frailty in these patients, allowing them to safely undergo curative surgery. Studies have shown that nutritious dietary supplements, exercise, and cognitive training can improve frailty status. Individually designed, multifactorial therapies administered by a multidisciplinary team consisting of physiotherapists, geriatricians, rehabilitation physicians, nurses, and nutritionists may improve frailty status and ultimately enhance outcomes following pancreatic surgery (41–43).

## Conclusion

The presence of preoperative frailty may lead to increased risk of short-term mortality and morbidity in patients undergoing pancreatic surgery. Frailty was not associated with major complications, readmission, or reoperations. However, results must be interpreted with caution owing to limited data and high inter-study heterogeneity, especially for frailty assessment tools. Future studies using standardized definitions of frailty are needed for robust conclusions.

## Data Availability

Publicly available datasets were analyzed in this study. The data used in the studies are available on the databases of PubMed, Embase, Scopus, and Web of Science. Further data on the analysis can be obtained from the corresponding author on reasonable request.

## Supplementary material

Supplementary Figure 1 Funnel plot for meta-analysis on all complications.

Supplementary Figure 2 Funnel plot for meta-analysis on CD ≥ 3 complications.

Supplementary Figure 3 Funnel plot for meta-analysis on readmission.

Supplementary Figure 4 Funnel plot for meta-analysis on short-term mortality.

Supplementary Figure 5 Sensitivity analysis for all complications.

Supplementary Figure 6 Sensitivity analysis for CD ≥ 3 complications.

Supplementary Figure 7 Sensitivity analysis for short-term mortality.
