# Supplementary figures and images for: Impact of frailty on outcomes of pancreatic surgery: a systematic review and meta-analysis
Source: Front Nutr. 2026 Mar 2;13:1706900. doi: 10.3389/fnut.2026.1706900 (PMC12989370; doi:10.3389/fnut.2026.1706900)

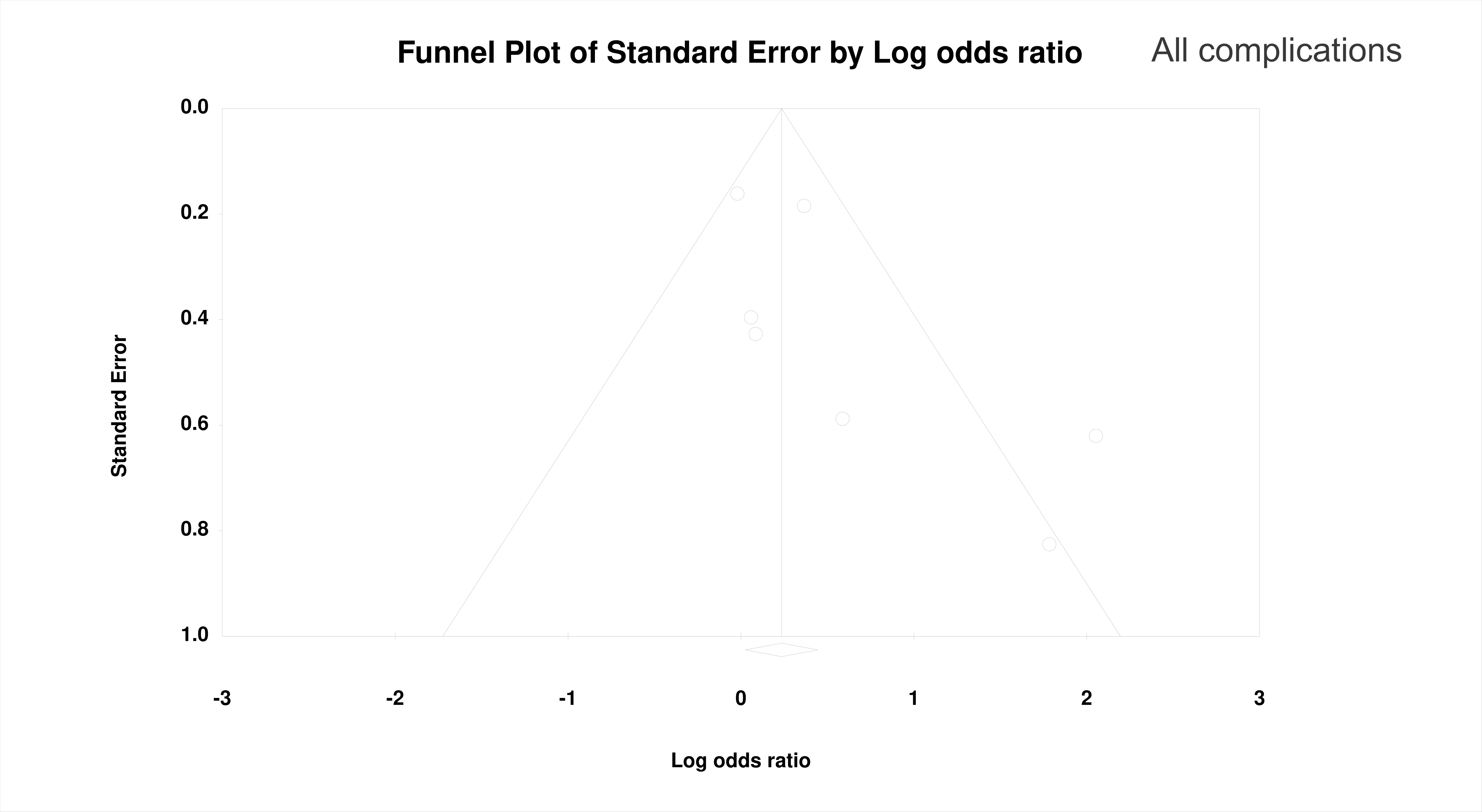

Supplement: Supplementary Figure 1 — Funnel plot for meta-analysis on all complications. [file Image_1.jpeg]

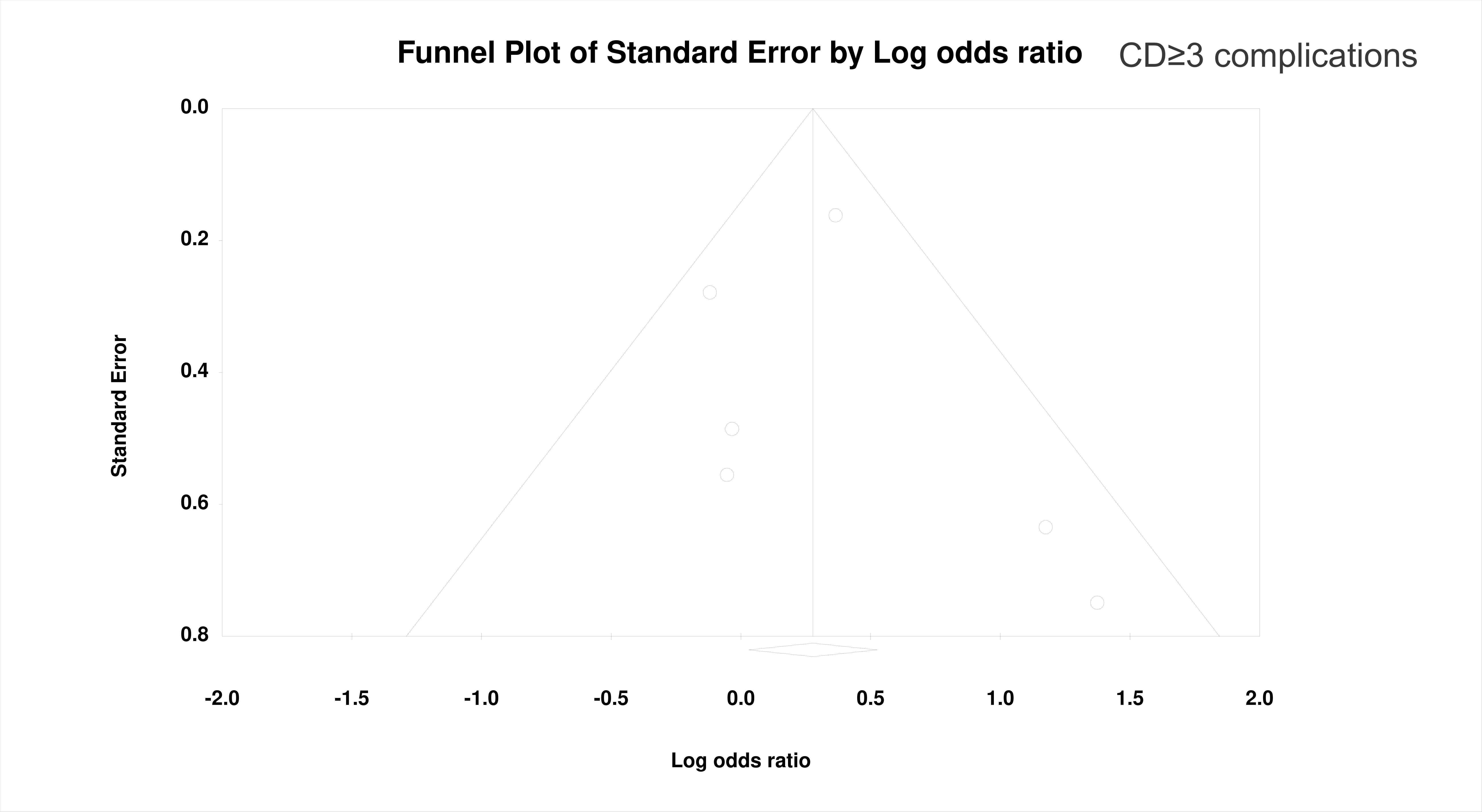

Supplement: Supplementary Figure 2 — Funnel plot for meta-analysis on CD ≥ 3 complications. [file Image_2.jpeg]

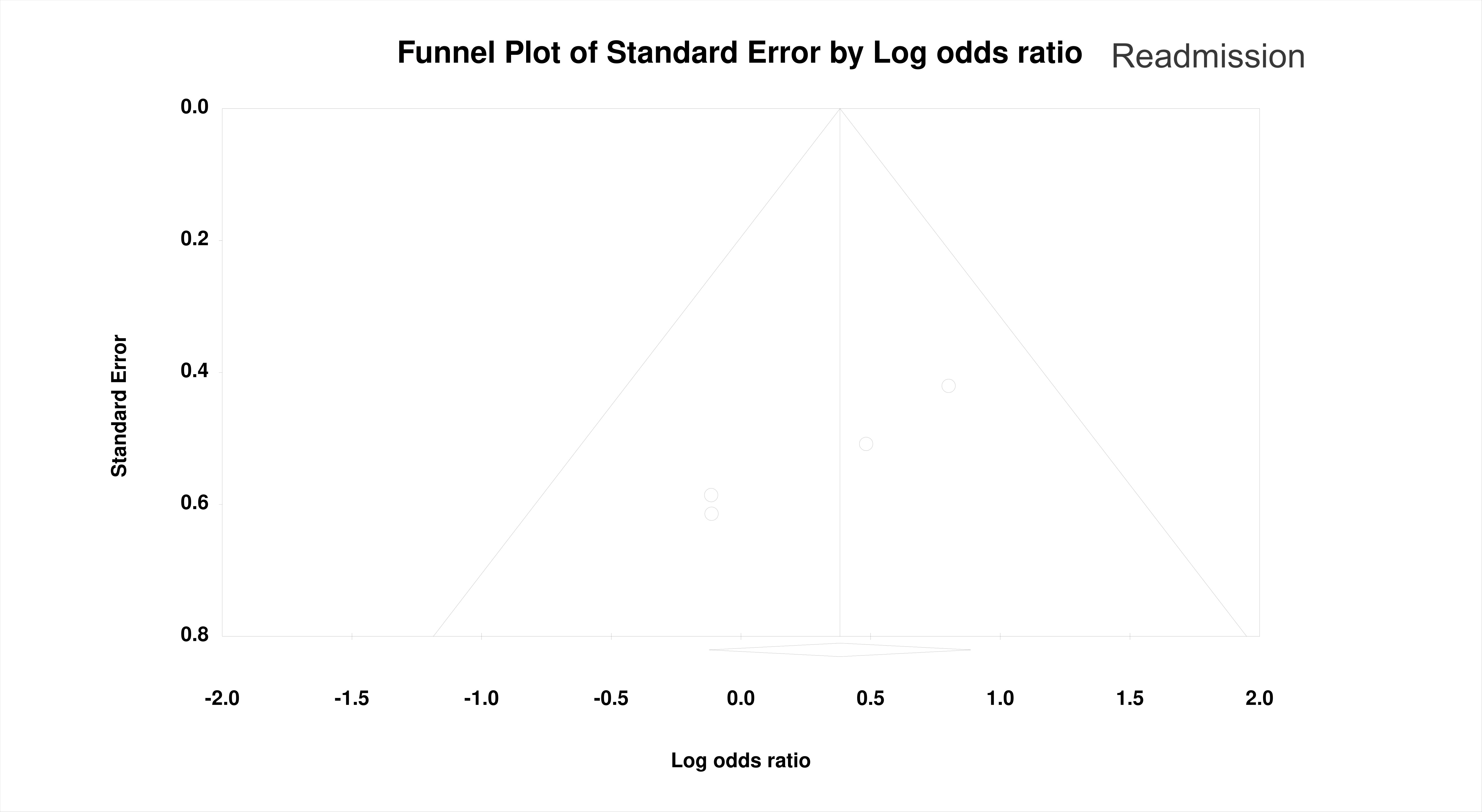

Supplement: Supplementary Figure 3 — Funnel plot for meta-analysis on readmission. [file Image_3.jpeg]

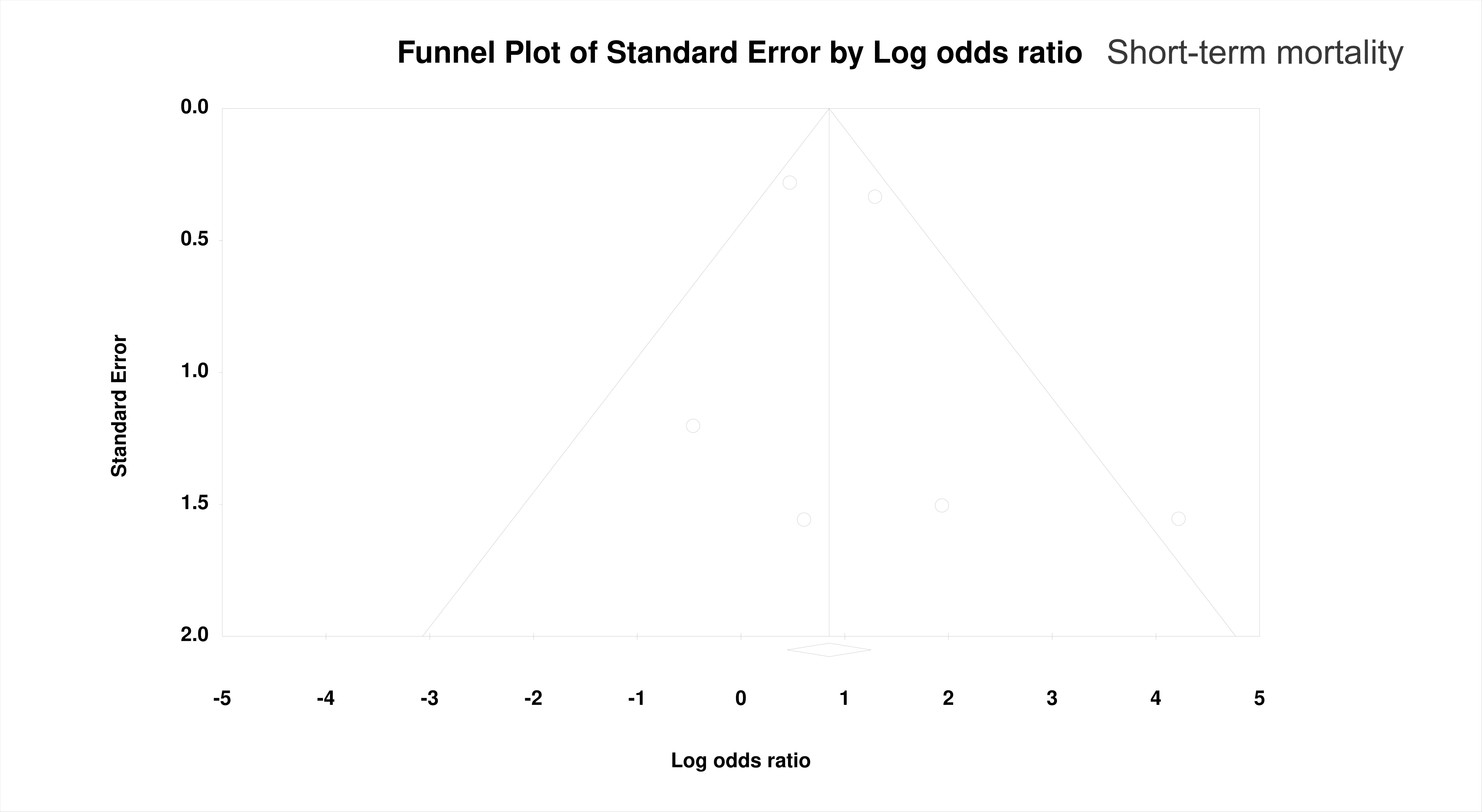

Supplement: Supplementary Figure 4 — Funnel plot for meta-analysis on short-term mortality. [file Image_4.jpeg]

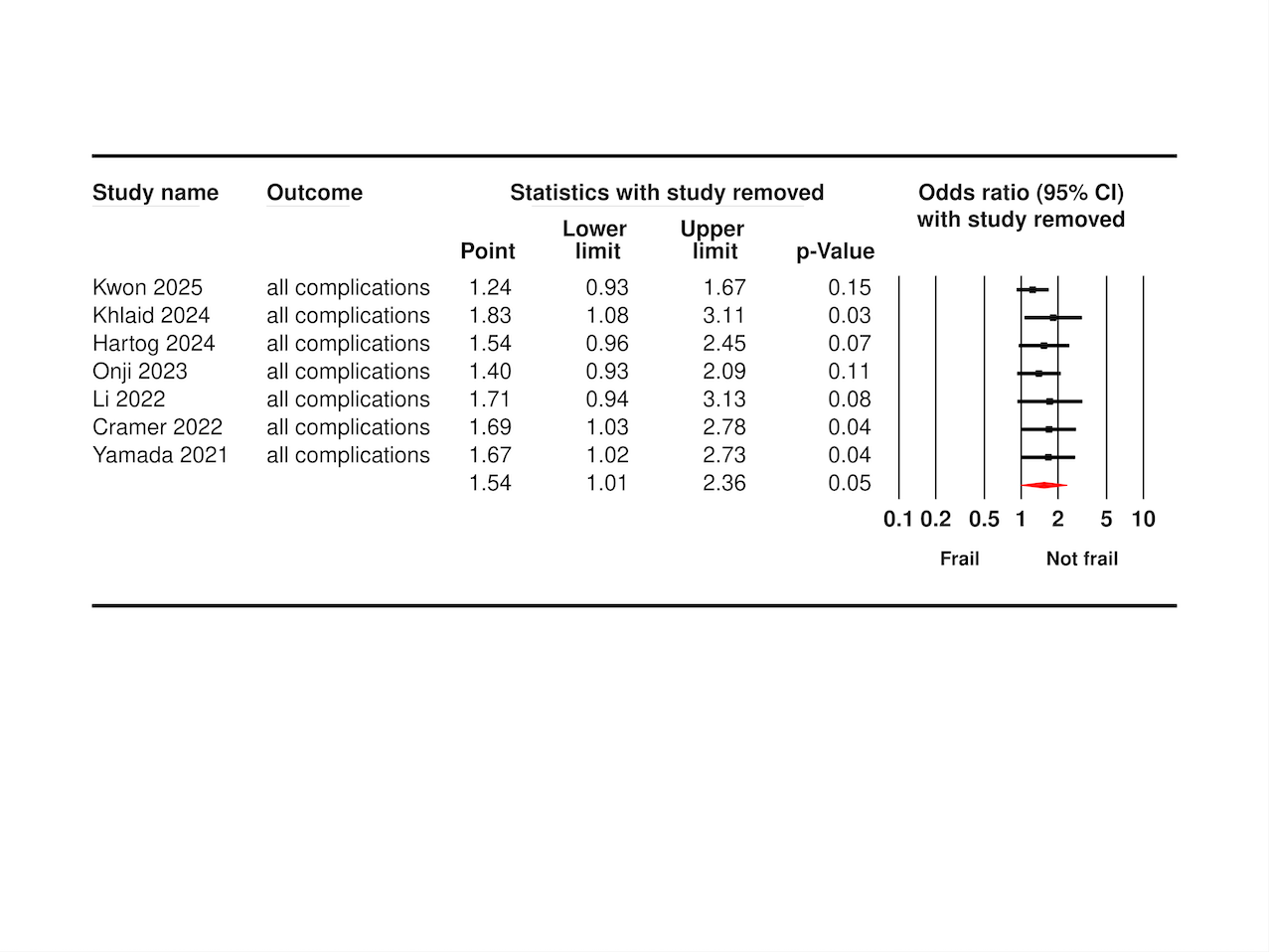

Supplement: Supplementary Figure 5 — Sensitivity analysis for all complications. [file Image_5.tiff]

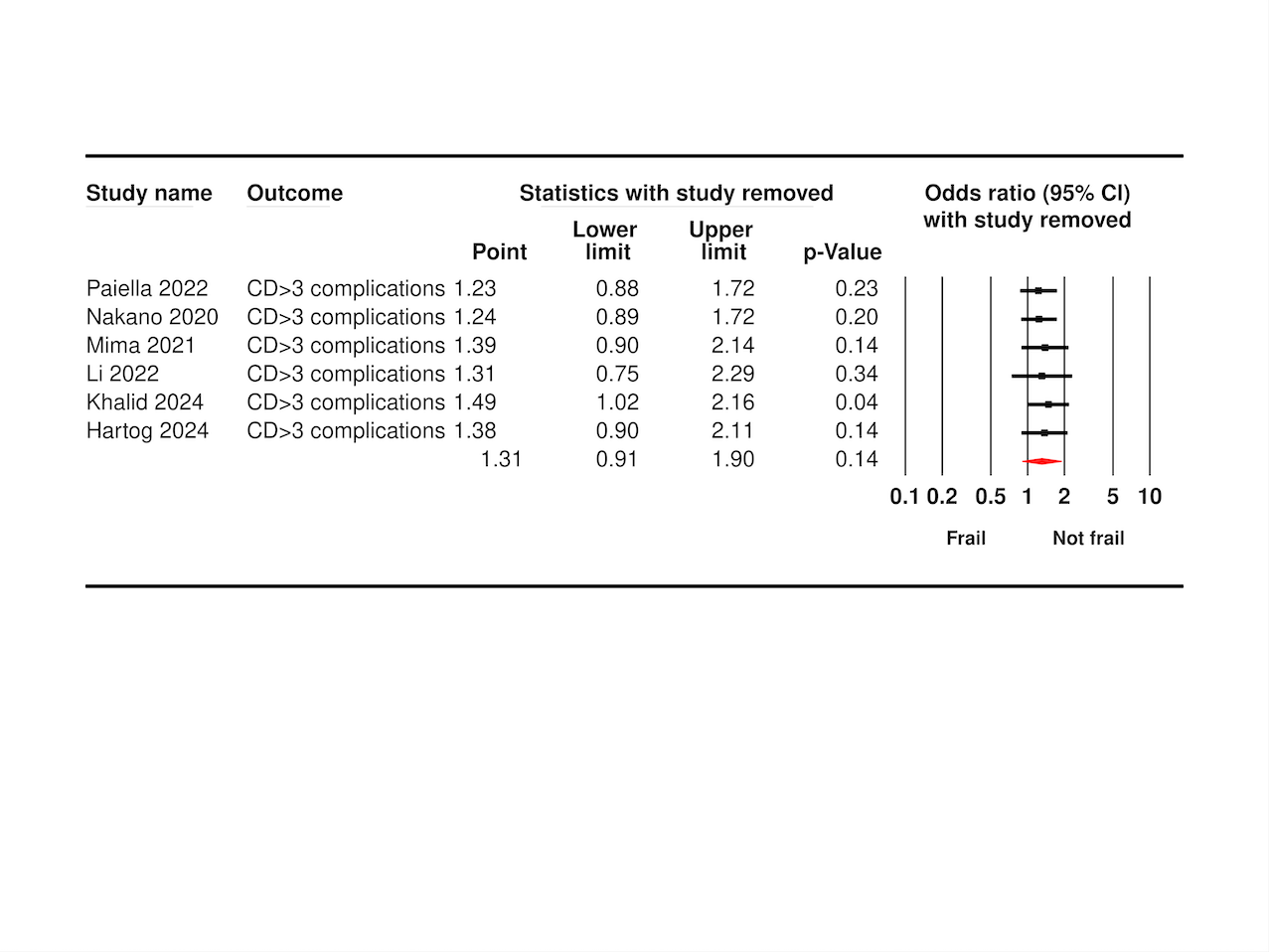

Supplement: Supplementary Figure 6 — Sensitivity analysis for CD ≥ 3 complications. [file Image_6.tiff]

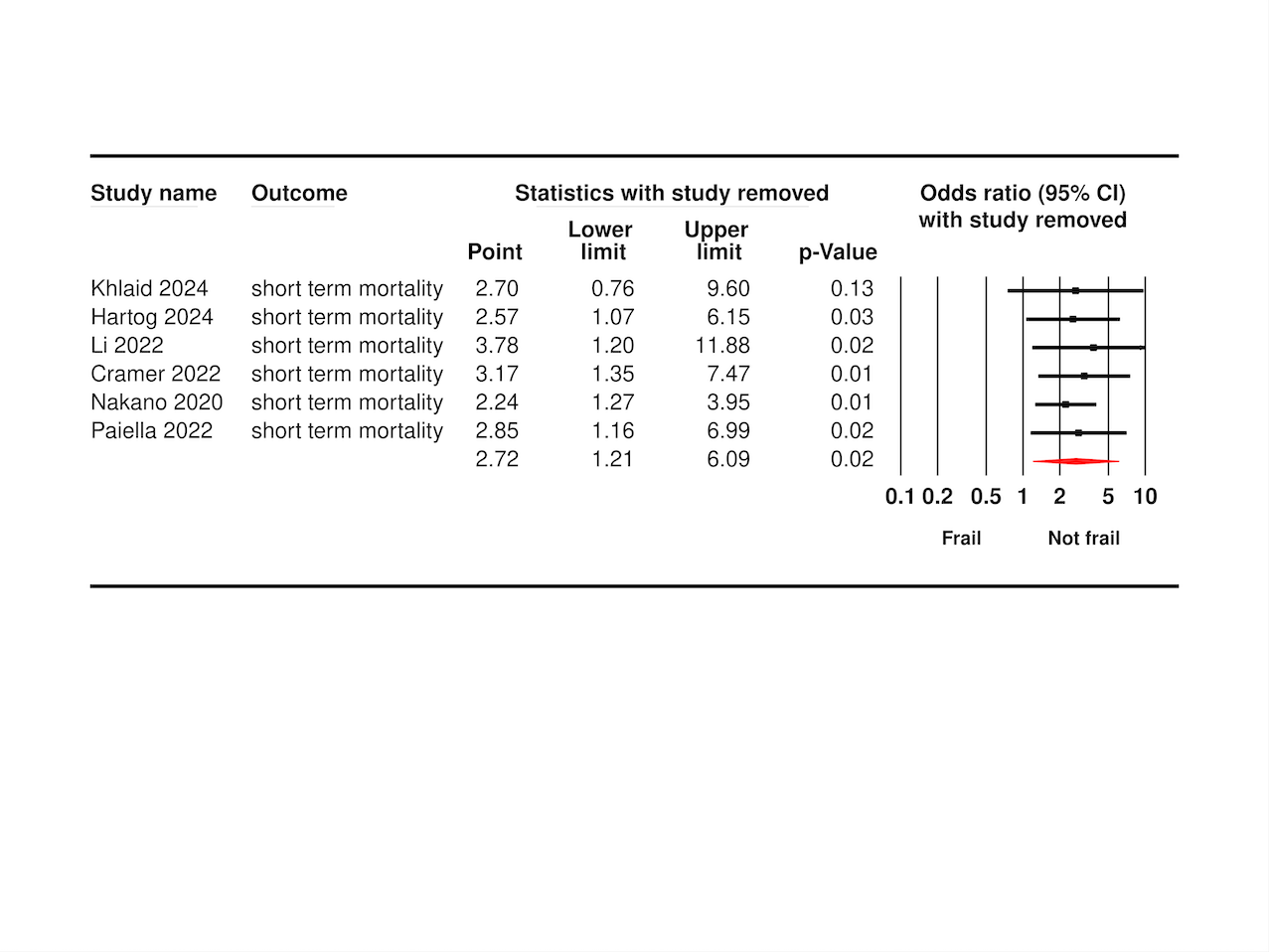

Supplement: Supplementary Figure 7 — Sensitivity analysis for short-term mortality. [file Image_7.tiff]
