# Supplementary material for: Impact of frailty on outcomes of pancreatic surgery: a systematic review and meta-analysis
Source: Front Nutr. 2026 Mar 2;13:1706900. doi: 10.3389/fnut.2026.1706900 (PMC12989370; doi:10.3389/fnut.2026.1706900)
Supplement: Supplementary file 8 [file Table_1.docx]

Supplementary Table 1: Search strategy of all databases

| Database | Search strategy |
| --- | --- |
| PubMed | (((((pancreatectomy) OR (pancreatic surgery)) OR (pancreatic resection)) OR (pancreaticoduodenectomy)) AND (((frail) OR (frailty)) OR (geriatric assessment))) AND ((((mortality) OR (complications)) OR (reoperation)) OR (readmission)) |
| Embase | 1. 'radical pancreatectomy'/exp OR 'radical pancreatectomy' OR (pancreatic AND surgery) OR (pancreatic AND resection) OR 'pancreaticoduodenectomy'  2. 'frailty'/exp OR 'frailty' OR frail OR 'geriatric assessment'  3. 'mortality'/exp OR 'mortality' OR 'complication' OR 'hospital readmission' OR 'reoperation'  4. #1 AND #2 AND #3 |
| Scopus | (TITLE-ABS-KEY-AUTH (pancreatectomy) OR (pancreatic surgery) OR (pancreatic resection) OR (pancreaticoduodenectomy)) AND (TITLE-ABS-KEY-AUTH(frail) OR (frailty) OR (geriatric assessment)) AND (TITLE-ABS-KEY-AUTH(mortality) OR (complications) OR (reoperation) OR (readmission)) |
| Web of Science | (((((pancreatectomy) OR (pancreatic surgery)) OR (pancreatic resection)) OR (pancreaticoduodenectomy)) AND (((frail) OR (frailty)) OR (geriatric assessment))) AND ((((mortality) OR (complications)) OR (reoperation)) OR (readmission)) |
